# Development and Application of Flue Gas Desulfurized Gypsum and Blast-Furnace-Slag-Based Grouting Material for Cracked Silty Mudstone

**DOI:** 10.3390/ma17235975

**Published:** 2024-12-06

**Authors:** Guangtao Yu, Hongyuan Fu, Qianfeng Gao, Ling Zeng, Jingcheng Chen, Chongsen Ma

**Affiliations:** 1 School of Traffic & Transportation Engineering, Changsha University of Science and Technology, Changsha 410114, China; yuguangtao163@163.com (G.Y.);; 2 National Key Laboratory of Green and Long-Life Road Engineering in Extreme Environment, Changsha 410114, China; 3 School of Civil Engineering, Changsha University of Science and Technology, Changsha 410114, China; 4 The Third Construction Co., Ltd. of China Construction Fifth Engineering Bureau, Changsha 410004, China

**Keywords:** silty mudstone, grouting material, FGDG, BFS, mechanical strength, continuous–discontinuous element method

## Abstract

The grouting technique is an efficient method for enhancing the stability of cracked slopes through the use of grouting materials. Conventional cement-based grouting materials are costly, energy-intensive, and environmentally damaging. Additionally, cement-hardening slurry is prone to cracks between the slurry and the rock. To address these issues, this study proposed an environmentally friendly grouting material composed of flue gas desulfurization gypsum (FGDG) and blast furnace slag (BFS) with sodium gluconate (SG) as the additive, especially designed for cracked silty mudstone slopes. The effects of different FGDG-to-BFS ratios and SG dosages on the setting time, fluidity, shrinkage, unconfined compressive strength (UCS), tensile strength, and shear strength parameters of hardened grouting slurries, as well as the interfacial bonding strength between silty mudstone and the hardened slurries, were investigated through laboratory tests. Subsequently, the improvement effects of cement-based material and the FGDG–BFS material on cracked silty mudstone were compared by mechanical tests. Finally, the performance of both types of grouting material on cracked silty mudstone slopes was analyzed by numerical simulations based on GDEM. The results demonstrated that the optimal FGDG-to-BFS ratio was 0.8:1, under which, the mechanical properties of the hardened FGDG–BFS slurries cured for 14 days exceeded those of the silty mudstone. The optimal dosage of SG was 0.4%, effectively prolonging the setting time of the slurry and improving the water resistance of the hardened slurries. The FGDG–BFS material exhibited a superior performance in repairing rock cracks compared to cement-based materials, with the damage patterns of the grouted specimens aligning with those of the intact specimens. This new grouting material effectively repaired existing cracks and prevented re-cracking at the interface between the grouting material and silty mudstone, thereby maintaining slope stability over a long period.

## 1. Introduction

Silty mudstone is widely distributed in the hot and humid areas of southern China [1,2]. Since its clay mineral content exceeds 50%, silty mudstone is susceptible to cracking under hot and humid climate conditions [3,4]. Cracks can reduce the shear strength of slopes and weaken their stability. Under the effect of humidity–heat cycles, the maximum width and depth of cracks in soft rock such as silty mudstone can reach 0.06–0.10 m and 1.0–1.5 m, respectively [5,6]. Grouting is one of the common and effective methods to repair cracked rock slopes [7]. Compared with the anchor and soil nail methods, the grouting method offers advantages such as a low cost, independence from geological conditions, and a shorter construction time. Meanwhile, since the slurry can be pressed into the interior of the slope by a grouting machine, grouted slopes achieve a better integrity [8,9]. In the whole process of the grouting method, the grouting material is the key factor affecting the effectiveness of the grouting repair [10].

Existing grouting materials can be classified into inorganic and organic types based on their molecular components [11]. Inorganic grouting materials include pure cement materials, cement-based materials, and water-glass-based materials [12,13]. Pure cement materials are characterized by simple raw materials, a low cost, good seepage resistance, and a high strength [14]. However, pure cement materials also have shortcomings, such as large particles, a long setting time, a slow rate of strength growth, easy segregation and precipitation, and a poor material stability [15]. These shortcomings limit the scope of the application of pure cement materials. To address these issues, researchers added clay, bentonite, and other inert materials to a pure cement slurry to obtain a cement-based material [16]. However, adding soil caused an increase in the setting time and a decrease in the strength of cement-based materials [17]. For this reason, researchers investigated water-glass-based inorganic grouting materials [18]. Compared with cement grouting materials, water-glass-based grouting materials have a higher strength and better impermeability [19]. Nevertheless, the shortcomings of water-glass-based grouting materials, such as poor abrasion resistance and easy cracking, limit their application [20].

To overcome the shortcomings of inorganic materials, professionals have started to apply organic-based grouting materials for the repair of cracked rock, such as acrylamide-based materials, polyurethane-based materials, and urea formaldehyde resin-based materials [21,22]. Compared with inorganic grouting materials, organic grouting materials have a better fluidity, stability, and impermeability, as well as an adjustable setting time [23]. However, organic grouting materials are more costly and polluting, which harms the environment and poses health risks to workers [24]. Organic grouting materials also have shortcomings, such as high equipment requirements and construction difficulties [25].

In China, industries such as iron and steel production, thermal power generation, and others discharge large amounts of fly ash (FA), blast furnace slag (BFS), flue gas desulfurization gypsum (FGDG), and other industrial solid wastes [26,27]. Among them, the annual output volume of FA is more than 120 Gt, with a comprehensive utilization rate of less than 20% [28]. The annual output volume of FGDG is about 0.43 Gt, with a comprehensive utilization rate of about 56%. The low utilization rates and high emissions of industrial solid waste cause significant waste accumulation, which not only occupies arable land, but also pollutes water and air, threatening the health of local residents and agricultural production [29,30].

Therefore, improving the utilization rate of industrial solid waste and reducing industrial waste accumulation have become key research areas. Duan et al. [31] used FA, calcium carbide slag (CCS), and FGDG as raw materials to prepare geopolymer grouting reinforcement materials. They found that the 28 d compressive strength of the grouting material could reach 20.5 MPa and the alkali activator could effectively improve the fluidity and setting time of the grouting material. Feng et al. combined FGDG with titanium slag and Portland cement to construct TS–FGD–OPC composites, which were found to be effective in improving the unconfined compressive strength and elastic modulus of the soil, and the pH of the cured soil was within the normal range [32]. Guo et al. prepared a high-performance bonding material consisting of 96% gypsum hemihydrate (HH) and 4% hard gypsum by calcining FGDG at 190 °C for 90 min. Compared with FGDG, the new material had a shorter setting time and higher mechanical strength [33]. Chen et al. used FGDG as a raw material and a hydrothermal method to prepare a multifunctional and high-value-added building material, calcium sulfate hemihydrate whisker (CSHW), which can be used for the rational disposal of FGDG [34].

In summary, the existing industrial solid-waste-based grouting materials are mainly prepared by replacing part of the cement with industrial solid waste to obtain industrial solid-waste–cement-based grouting materials with the same strength as traditional cement-based grouting materials. This method has two drawbacks [35,36]. First, the amount of cement used in the above grouting materials generally exceeds 60%, which does not completely replace the cement and fundamentally solves the pollution problem of cement materials. Secondly, the main use of the above materials is to reinforce foundations, bridges, and tunnels, etc., and their strength is much higher than that of soft rocks such as silty mudstone. If the above materials are used to reinforce soft rocks, the interface between the rock and the grouting material is prone to cracking, weakening the repair effect [37].

The aim of this study is to develop a new environmentally friendly industrial solid-waste-based grouting material (FGDG–BFS material). Compared with traditional cement-based grouting material and the existing FGDG-based grouting material, the physical properties of the FGDG–BFS material are more similar to those of silty mudstone. After grouting with the FGDG–BFS material, the integrity of cracked silty mudstone is higher, which is more useful for its repair, preventing secondary cracking, and improving the stability of the slope. Meanwhile, the preparation and application of this FGDG–BFS material can effectively improve the utilization rate of industrial solid waste to improve the stability of grouted cracked slopes and increase the utilization of industrial solid wastes. Firstly, the optimal ratio of FGDG–BFS material was obtained, and its advantages were revealed by laboratory tests. Then, numerical analysis software (GDEM) was employed to investigate the effectiveness of the FGDG–BFS material in improving the stability of cracked silty mudstone slopes. The findings could offer valuable guidance for the reinforcement of easy-cracking silty mudstone slopes.

The main innovations of this paper are as follows:

1. This paper mainly develops a new environmentally friendly industrial solid-waste-based grouting material (FGDG–BFS material) to improve the stability of grouted cracked slopes and increase the utilization of industrial solid wastes.
2. Numerical analysis software (GDEM) was employed to investigate the effectiveness of the FGDG–BFS material in improving the stability of cracked silty mudstone slopes.

## 2. Materials and Test Scheme

### 2.1. Materials

#### 2.1.1. Industrial Solid Waste

**Figure 1:**
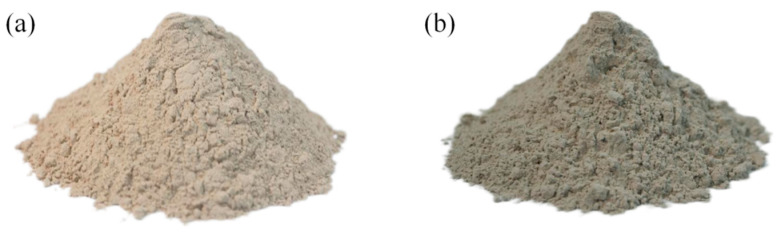
Photographs of two industrial solid wastes: (**a**) FGDG and (**b**) BFS.

The FGDG powder and BFS powder used in this study were sourced from Hebei Shengyun Mineral Factory (China). The FGDG powder primarily consisted of CaSO_4_·2H_2_O, showing a desirable flowability and high strength after hardening with minimal water consumption. BFS is a byproduct of the ironmaking process, comprising residues from ore veins, ash from fuel, and non-volatile components in the solvent. Figure 1 shows photographic images of the FGDG powder and BFS powder. FGDG is light brownish yellow powder, the particle size varies, with shapes of mostly rectangular bars, and the surface has no micropores. The BFS powder is dark brown, with a smooth particle surface and irregular particle morphology. To accelerate the hydration of FGDG–BFS and improve its activeness, NaOH solution was chosen as the alkaline exciter in this study. Meanwhile, NaOH solution with a mass fraction of 10% was used to avoid water affecting the test results and ensure the safety of the test operation.

The basic physical parameters of the FGDG powder and BFS powder are shown in Table 1. The specific surface areas of the FGDG powder and BFS powder were 132 m^2^·kg^−1^ and 412 m^2^·kg^−1^, respectively. Their average particle sizes were 44.44 μm and 12.09 μm, with apparent densities of 2.30 g·cm^−3^ and 2.90 g·cm^−3^. X-ray fluorescence analysis (XRF) detected 31.12% CaO, 44.44% SO_3_, and a 17.8% crystalline water content. The main components of the blast furnace slag powder were CaO (43.19%), SiO_2_ (25.52%), Al_2_O_3_ (12.60%), and MgO (15.21%), as shown in Table 2. Due to the FGDG powder mainly comprising gypsum and the BFS powder being amorphous without crystalline phases, X-ray diffraction analysis (XRD) did not detect specific mineral phases in either material.

#### 2.1.2. Additives

The poor water resistance and short setting time of gypsum-based grouting materials are important factors that limit the scope of their application. Therefore, in this study, SG was used as an additive to achieve the purpose of improving the water resistance and setting time of BFS–FGDG solid waste grouting. Sodium gluconate, an organic substance with the chemical formula C_6_H_11_NaO_7_, is highly soluble in water and appears as a white crystalline powder. The pH of SG is neutral, and its melting point is 206 °C. SG is an environmentally friendly material, which can be rapidly and completely degraded by ordinary biochemical treatment, causing no pollution to the natural environment.

#### 2.1.3. Silty Mudstone

The test material was collected from a highly weathered silty mudstone slope in the Daijiaping Formation of Middle Devonian in Dongyang Town, Hunan Province, China. The silty mudstone consisted of mineral particles, intergranular adhesion and pores, microcracks, and water and gas. The main minerals of the silty mudstone, as revealed by X-ray diffraction (XRD), were quartz (46.0%), illite (28.8%), montmorillonite (9.8%), feldspar (8.3%), and chlorite (7.7%). The main chemical components of the silty mudstone were SiO_2_, Al_2_O_3_, Fe_2_O_3_, K_2_O, and MgO. The sum of the mass fractions of SiO_2_ and Al_2_O_3_ accounted for approximately 80% of all chemical components in the silty mudstone. Silty mudstone is highly susceptible to cracking under hot and humid environmental conditions. Cracking can lead to a reduction in the strength of silty mudstone and increase the risk of the instability of silty mudstone slopes.

### 2.2. Grouting Slurry Preparation Schemes and Test Schemes

#### 2.2.1. Grouting Slurry Preparation Schemes

Firstly, a sufficient amount of distilled water was weighed according to a water–solid ratio of 1:2, and sufficient amounts of FGDG, BFS, C_6_H_11_NaO_7_ powder, and NaOH solution were weighed according to the optimal ratio and dosage, and put beside the mixer as spares. After that, distilled water, FGDG, and BFS were poured into the mixer in turn, and the mixer was started. Then, the C_6_H_11_NaO_7_ powder and the NaOH solution were poured into the mixer. The above materials were stirred well in the blender for 5 min to ensure that the materials were well mixed.

#### 2.2.2. Test Schemes

Silty mudstone slopes are prone to cracking and shear sliding, leading to slope collapse. The material interface strength and UCS are important indexes affecting the strength, integrity, and toughness of the rock after grouting. Therefore, this study determined the optimal BFS-to-FGDG ratio by testing the above four strength indexes. Subsequently, setting time tests, flow tests, water resistance tests, and shrinkage tests were conducted to determine the optimal dosage of SG. Finally, the improvement effects of the materials were explored by comparing the strength changes in the cracked silty mudstone before and after grouting using cement-based grouting materials and FGDG–BFS materials.

#### 2.2.3. Mechanical Tests of FGDG–BFS Hardened Slurries

**Figure 2:**
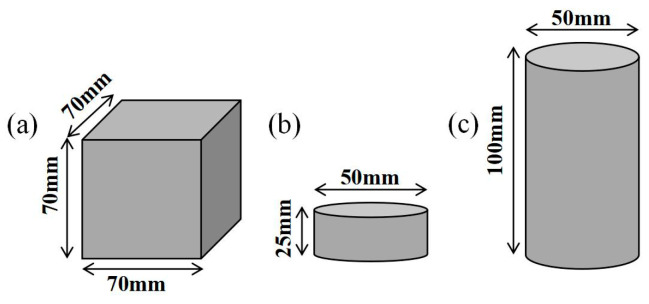
Three types of specimens: (**a**) Direct shear test specimen (**b**) Brazilian splitting test specimen (**c**) Unconfined compressive test specimen.

Through numerous pre-tests, the optimal water–solid ratio of the BFS–FGDG grouting material was determined to be 1:2. To determine the optimal BFS-to-FGDG ratio, five ratios (0:1, 0.2:1, 0.4:1, 0.6:1, 0.8:1, and 1:1) were designed based on the preliminary pre-tests, as shown in Table 3. The hardened slurries included the following three types of specimens: cubic specimens with a side length of 70 mm, cylindrical specimens with a diameter of 50 mm and height of 25 mm, and cylindrical specimens with a diameter of 50 mm and height of 100 mm (Figure 2). All hardened slurries were cured under standard curing conditions for 14 days. Unconfined compressive tests, Brazilian splitting tests, direct shear tests, and interface strength tests were carried out for different ratios of the hardened slurries. The test steps were strictly in accordance with American rock and cement testing standards [38,39,40]. To diminish test errors, each group of tests was repeated five times, the error values in the test results were eliminated, and the remaining values were averaged later. The detailed mechanical tests steps are shown as follows:

(1) Unconfined compressive tests: The specimens, as shown in Figure 2c, were positioned on a universal testing machine, ensuring that the centerline of the specimens and the centerline of the loading end coincided. The strain control method was used to apply load to the specimen, and the loading rate was set at 0.002 mm·s^−1^.
(2) Brazilian splitting tests: The specimens, as shown in Figure 2b, were positioned on the universal testing machine and fixed to ensure that the centerline of the specimen coincided with the loading direction. The loading speed of the test was 0.1 mm·min^−1^, and the applied load was 600 kN. Equation (1) was used to calculate the tensile strength of the specimens.
  (1) $$\sigma_{t}=\frac{2P}{\pi DB}$$
  where *σ*_t_ is the tensile strength; *P* is the peak load; *D* is the specimen diameter; and *B* is the specimen thickness.
(3) Direct shear tests: The specimens shown in Figure 2a were positioned in the shear box, and the shear box was fixed afterwards. A normal force was applied to the specimen and four normal forces were set, as follows: 200 kN, 400 kN, 600 kN, and 800 kN. Then, shear force was applied to the specimen using the shear box displacement. When the specimens were destroyed, the test results were processed and the cohesive force and the internal friction angle of the specimens were obtained.
(4) Interface strength tests: The FGDG–BFS grouting material slurry was poured into the upper part of the mold with the silty mudstone as the substrate and cured for 14 days. After that, epoxy resin was applied on the standard block and bonded on the upper part of the grouting material hardened slurries. Then, the tensile test was started with a tensile speed of 5 mm·min^−1^. When the grouting material hardened slurries were detached from the silty mudstone, the tests were completed and the test data were recorded.

#### 2.2.4. Basic Engineering Characterization Tests of FGDG–BFS Material Hardened Slurries

We conducted pre-tests and identified the following five SG dosages: 0%, 0.1%, 0.2%, 0.3%, and 0.4%. To determine the optimal SG dosage, flow tests, setting time tests, water resistance tests, and shrinkage tests were carried out for FGDG–BFS materials with different SG dosages. The test steps strictly followed the American cement testing standard [40]. To avoid test errors, each group of tests was repeated five times, the error values in the test results were eliminated, and the remaining values were averaged. The detailed basic engineering characterization tests steps are shown as follows:

(1) Flow tests: The conical bucket was placed on a graduated glass plate and the FGDG–BFS material slurry was poured into the conical bucket. After that, the conical bucket was lifted up, the maximum diameters of the slurry in two directions perpendicular to each other after 30 min were measured, and the average value of the two values was taken to be recognized as the flowability of the grouting material.
(2) Setting time tests: The FGDG–BFS material slurry was poured into the mold, and after 2 h, the resistance value of the slurry began to be measured every 30 min. When the resistance value reached 0.7 MPa, this time was the final setting time of the slurry.
(3) Water resistance tests: The FGDG–BFS material hardening slurries were completely soaked in a thermostat for 7 days at a temperature of 25 °C. The UCS of the specimens was measured and compared to the UCS of the specimens submerged for 7 days. Afterwards, the UCS of the specimens soaked for 7 days was measured and compared with the UCS of the unsoaked specimens, and the value of the division between the two was the softening factor.
(4) Shrinkage tests: The volumes of the FGDG–BFS material hardening slurries cured for 14 days were measured and compared with the volume of standard specimens. The ratio of the difference between the two and the volume of the standard specimen was the shrinkage rate of the material.

#### 2.2.5. Mechanical Tests of Grouted Silty Mudstone

To investigate the improvement effect of the FGDG–BFS material on cracked silty mudstone, an unconfined compressive test was carried out on cracked silty mudstone grouted with FGDG–BFS material. The size of the prefabricated cracks was 60 mm wide and 3 mm long. The prefabricated cracks included the following five angles: 0°, 30°, 45°, 60°, and 90°. Silty mudstone specimens were cylindrical specimens with a 50 mm diameter and 100 mm height. The test equipment was a WED600 universal testing machine (Jinan Fangyuan Testing Machine Co., Ltd., Jinan, China). The test steps were strictly in accordance with American rock testing standard [38].

**Figure 3:**
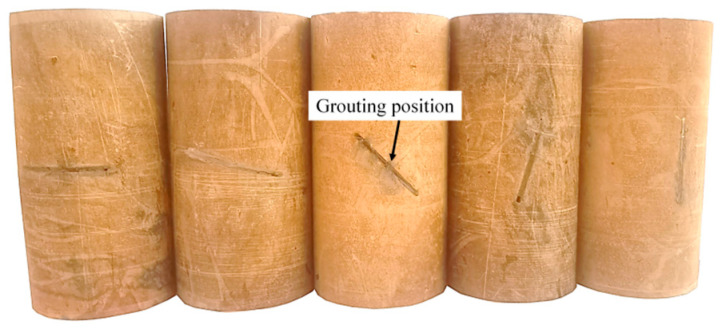
Grouted silty mudstone specimens (grouting time 10 min; curing time 14 days).

During the tests, the grouted silty mudstone specimens, which were cured for 14 days, were first placed vertically in a thermostat containing distilled water. The temperature of the thermostat was 25 °C and the distilled water was used to completely soak the specimens. The following six soaking times were set: 0 day, 5 days, 10 days, 15 days, 20 days, 25 days, and 30 days. In this study, the proportion of the cement-based material was C30 cement–sand–water = 1:2:0.4. Chen et al. [41] found that rocks with 45° cracks had the lowest UCS, so silty mudstone specimens with 45° cracks were used as the test specimens for the water resistance test. When the setup soaking time was reached, the specimens were removed from the thermostat box and the surface of the specimens was gently wiped clean. After that, UCS tests were carried out on the soaked specimens, and the UCS values of the unsoaked specimens were compared to investigate the water resistance of the grouted silty mudstone. The grouted specimens are shown in Figure 3.

## 3. Results Analysis

### 3.1. Mechanical Characteristics of Hardened Slurries

**Figure 4:**
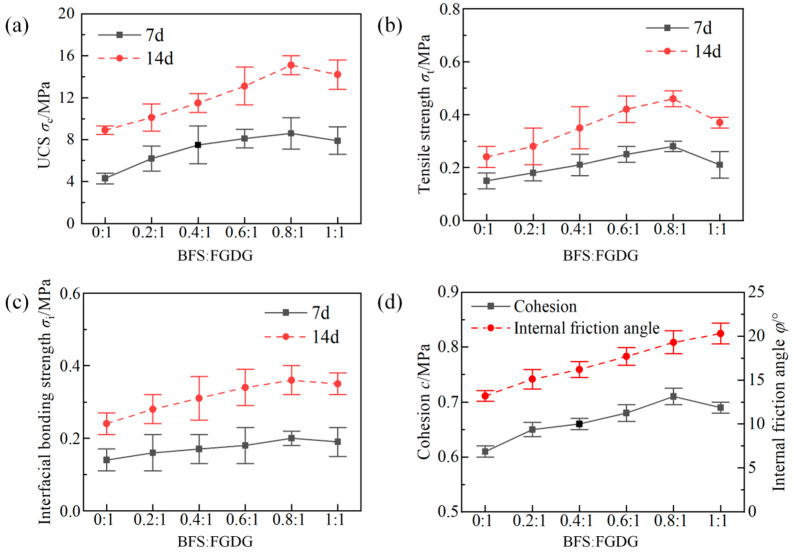
Mechanical properties of FGDG–BFS hardened slurries: (**a**) UCS; (**b**) tensile strength; (**c**) interfacial bonding strength; and (**d**) direct shear strength parameters.

Figure 4a,b show the change curves of the UCS and tensile strength of the hardened slurries with different FGDG-to-BFS ratios. The UCS and tensile strength of the hardened slurries at 7 d and 14 d showed a trend of increasing and then decreasing as the proportion of BFS increased. When the FGDG-to-BFS ratio was 0.8:1, the UCS and tensile strength of the hardened slurries reached a maximum of 15.6 MPa and 0.47 MPa, respectively. Figure 4c,d show the shear strength parameters of the hardened slurries and interfacial bond strength between the hardened slurries and silty mudstone with an increase in the ratio of FGDG to BFS. With an increase in the proportion of BFS, the agglomerate cohesion showed an increasing and then decreasing trend, while the internal friction angle gradually increased.

The strength of the grouting material hardened slurries was primarily due to the formation of a dense crystalline body structure in the hydration reaction of the FGDG slurries, which was mainly composed of gypsum dihydrate compounds. Another source of strength of the hardened slurries was the hydration reaction of BFS in an alkaline environment. The CaO, Al_2_O_3_, and SiO_2_ in the blast furnace slag generated a large number of calcium sulfoaluminate and calcium silica-aluminate gels under alkaline conditions, which increased the strength of the hardened slurries. Since the hydration process of the BFS powder enhanced the strength of the hardened slurries to a greater extent than the hydration of FGDG, the strength increased with the proportion of BFS powder. However, when the BFS exceeded the optimal ratio (0.8:1), due to the low viscosity of BFS, it could not compensate for the loss of strength caused by the decrease in the output of FGDG powder. Therefore, when the BFS–FGDG material exceeded the optimal ratio, the strength of the hardened slurries decreased. Furthermore, the hydration of BFS powder and FGDG powder caused a large number of gelatinous large particles to form inside the hardened slurries, increasing the internal friction angle.

### 3.2. Basic Engineering Properties Tests

**Figure 5:**
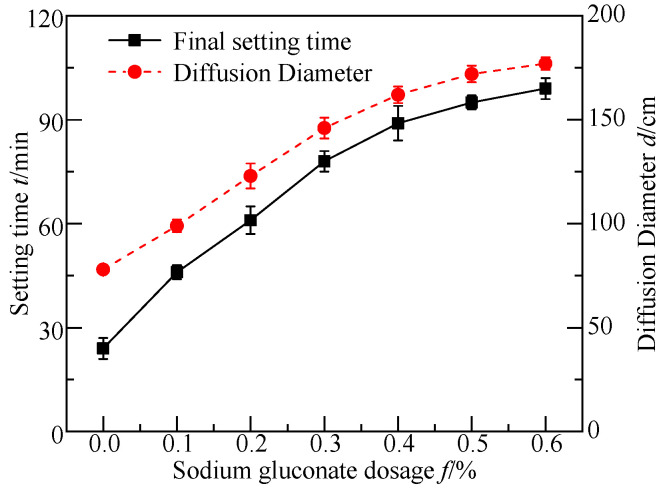
Setting time and flowability curves of FGDG–BFS materials with different dosages of SG.

**Figure 6:**
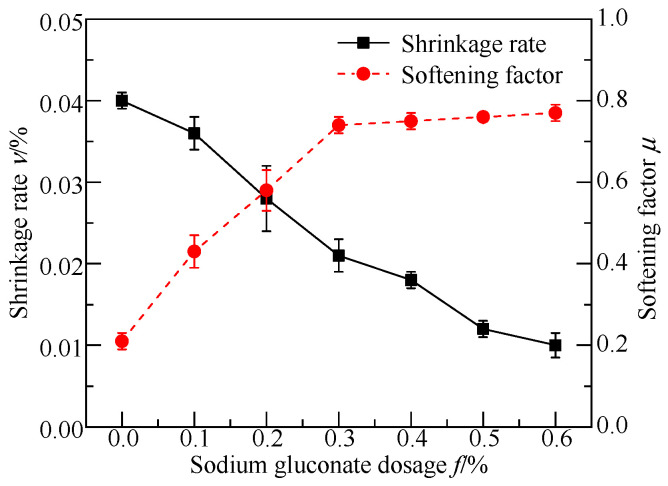
Shrinkage and softening factor curves of FGDG–BFS material with different SG dosages.

Figure 5 and Figure 6 show the change curves of the basic engineering properties of the FGDG–BFS material with different dosages of SG. It was found that the shrinkage of the grouting material decreased with an increase in the SG dosage. The softening factors, setting times, and diffusion diameters of the grouted materials increased initially and then gradually leveled off with an increase in the dosage of SG. The lowest dosages of the initial SG when the above parameters reached the leveling off state were 0.3%, 0.4%, and 0.4%, respectively.

Because SG adsorbed Ca^2+^ dissolved by the initial hydration of BFS and FGDG, it formed a molecular film on the surface of BFS and FGDG. This molecular film reduced the contact points between the BFS and FGDG mineral particles, weakened the inter-particle bridging, and hindered the coalescence of the BFS and FGDG particles. Additionally, the hydroxyl group (-OH) in the SG molecule formed hydrogen bonds on the surfaces of the BFS and FGDG particles, preventing their hydration. Therefore, SG effectively increased the setting time and flowability of the BFS–FGDG material.

SG functioned as a water reducer, effectively reducing the water–solid ratio and porosity of the hardened slurries, thereby improving densification. Meanwhile, SG combined with water to form a gel, avoiding the contraction of the hardened slurries due to water loss. The gel also prevented water from entering the hardened slurries, which could cause them to soften. Thus, the shrinkage of the hardened slurries decreased continuously with an increasing SG dosage. When the SG dosage was less than 0.4%, the softening factor of the hardened slurries increased with an increase in the SG dosage. However, when the SG dosage exceeded 0.4%, the BFS and FGDG material experienced increased inhibition, leading to product content with a lower hydration. Moreover, the softening coefficients of the hardened slurries were approximately equal at the SG dosages of 0.5% and 0.4%, as the excessive SG gel supplemented the hardened slurries’ resistance to external water. According to the American cement grouting material standard [42], the shrinkage rate of the grouting material should be less than 0.02%. Considering the flowability, setting time, and softening factor of the grouting material, the optimal SG dosage was determined to be 0.4%.

### 3.3. Improvement Effect

#### 3.3.1. FGDG–BFS Material Grouted Specimens

(1) *Unconfined compressive strength*

**Figure 7:**
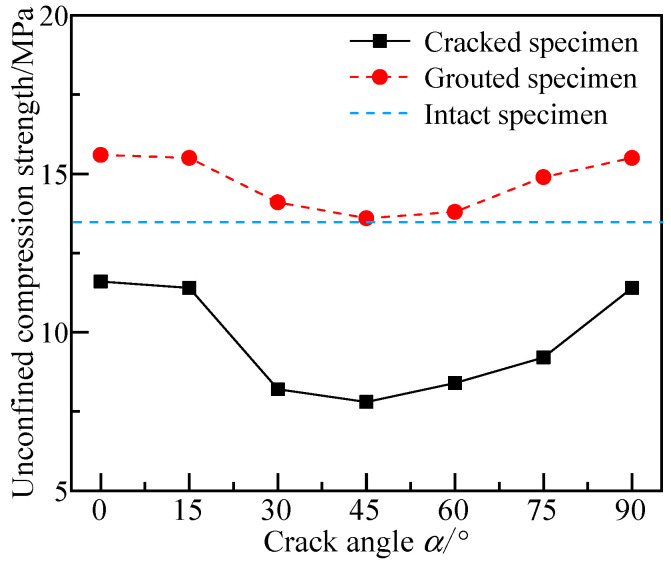
Variation in UCS of specimens with crack angle.

Figure 7 shows the comparison of the UCS of the prefabricated crack specimens, grouted specimens, and intact specimens. The UCS of the cracked silty mudstone specimens decreased and then increased with an increase in the crack angles. Specimens with a crack angle of 45° had the lowest UCS, with a reduction of 42.6% compared to the intact specimens. The FGDG–BFS material effectively improved the UCS of the prefabricated crack silty mudstone. The UCS of the grouted specimens exhibited a trend of decreasing and then increasing with the crack angle. The grouted specimen with a crack angle of 45° had the lowest UCS of (12.6 MPa), with the largest strength increase of 42.6%. The grouted specimens with a crack angle of 0° had the highest UCS of 14.6 MPa and the lowest strength increase of 14.7%. This was because the damage pattern of the specimen with a crack angle of 45° was primarily slide shear damage, making it more prone to damage compared with the specimens with other crack angles. Although the FGDG–BFS material effectively filled the cracks and the shrinkage rate of the FGDG–BFS material hardened slurries was close to that of the silty mudstone specimens, a gap still existed. Therefore, the grouted specimens with a crack angle of 45° were still more susceptible to slip shear damage than those with other crack angles. The specimens with crack angles of 0° and 90° mainly exhibited splitting damage along the central axis. The UCS of the FGDG–BFS material hardened slurries was higher than that of the intact specimens, which effectively improved the resistance of the specimens. Therefore, the UCS of the grouted specimens showed a U-shaped distribution with increasing crack angles. The difference between the UCS of the FGDG–BFS material hardened slurry and that of the silty mudstone was 8.46%. Xu et al. [43] prepared a high-performance grouting material using ultrafine cement and active mineral composite additives, and the difference between the UCS of the above grouting material and the UCS of the target rock (mudstone) was 108.50%. Yu et al. [44] used cement-silicate grouting material to repair the cracked mudstone, and the UCS difference between the above grouting material and the target rock (mudstone) was 31.87%. The above results prove that the mechanical properties of the FGDG–BFS material hardened slurry were closer to the target rock, which is more conducive to avoiding the generation of secondary cracks at the interface between the rock and the slurry material, which would weaken the repair effect.

(2) *Damage patterns*

**Figure 8:**
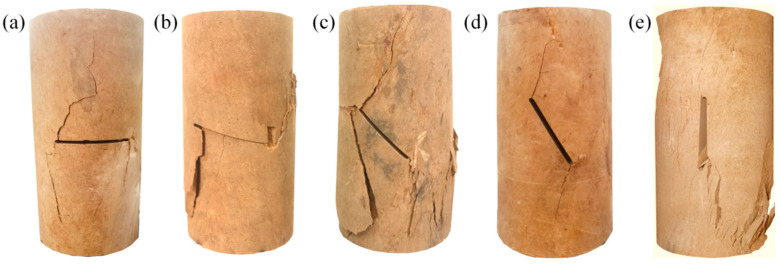
Damage patterns of specimens with prefabricated crack: (**a**) 0° (**b**) 30° (**c**) 45° (**d**) 60° (**e**) 90°.

**Figure 9:**
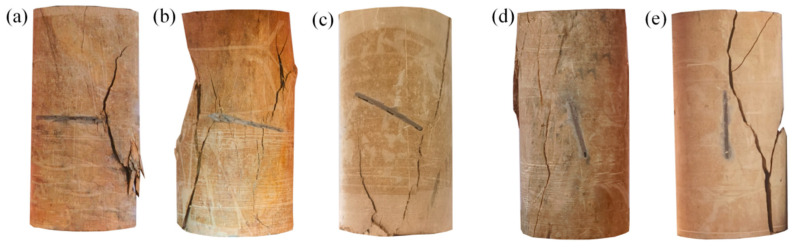
Damage patterns of grouted specimens: (**a**) 0° (**b**) 30° (**c**) 45° (**d**) 60° (**e**) 90°.

Figure 8 and Figure 9 show the damage patterns of the prefabricated crack and grouted specimens. From Figure 8, it can be seen that the crack extension path of the silty mudstone containing prefabricated cracks followed the direction of the prefabricated cracks. This was because the existence of prefabricated cracks caused strength damage inside the specimens. According to the fracture mechanics theory, the tip of the prefabricated crack was more likely to generate a tip stress concentration under the action of an external load. The crack generated at the tip of the crack first, and then gradually developed along the direction of the prefabricated crack, eventually penetrating the specimens.

The grouted specimens with crack angles of 0° and 30° did not follow the main path of crack development along the prefabricated crack, although the crack development path passed through the prefabricated crack. The crack development paths of specimens with crack angles of 45°, 60°, and 90° did not pass through the prefabricated crack at all. This demonstrated that the FGDG–BFS material effectively resolved the stress concentration problem at the tip of the prefabricated crack, making the cracked silty mudstone behave like an intact specimen. It was proven that the FGDG–BFS material was effective in repairing the cracks.

#### 3.3.2. Improvement Effects of Different Grouting Materials

(1) *Water resistance*

**Figure 10:**
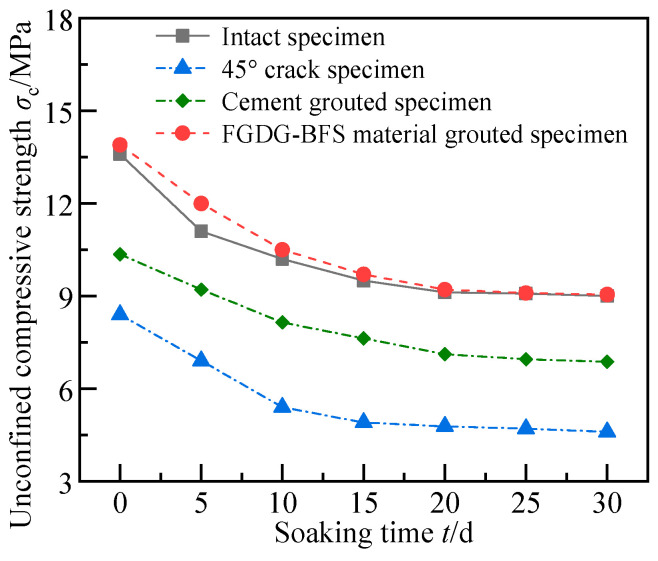
Variation in UCS of different specimens with water soaking time.

Figure 10 shows the variation curves of the UCS with soaking time for the intact specimens, the specimens with a 45° crack angle, and the grouted specimens. It shows that the UCS of the four specimens, in descending order, was as follows: the specimen grouted with the FGDG–BFS material, the intact specimen, the specimen grouted with cement-based material, and the specimen with a 45° crack. The UCS of the four types of specimens decreased and then leveled off with an increasing soaking time. The greatest final reduction in UCS was observed for the prefabricated crack specimen (45.2%), with final reductions of 33.8%, 34.49%, and 31.8% for the intact specimen and the specimens grouted by cement-based materials and the FGDG–BFS material, respectively. The soaking times at which the UCS of the intact specimen, the specimen with a 45° crack, and the specimens grouted by cement-based materials and the FGDG–BFS material approached plateauing were 20 d, 15 d, 20 d, and 20 d, respectively.

This was because, compared with cement-based materials, the difference between the shrinkage and mechanical properties of the FGDG–BFS material and silty mudstone was smaller, and the interfacial bond strength was greater, so the improvement effect of the FGDG–BFS material was better. The area of contact with moisture was larger in the prefabricated crack specimens, and the moisture infiltrated into the specimens quickly and uniformly along the prefabricated cracks. As a result, the UCS of the prefabricated crack specimens eventually decreased the most and slowed down the fastest. Since the softening factor and densification of the FGDG–BFS material hardened slurries were higher than those of the silty mudstone, the FGDG–BFS material effectively prevented water from softening the specimens along the prefabricated cracks. The above results demonstrate that FGDG–BFS material could have a sustained remediation role.

(2) *Damage patterns*

**Figure 11:**
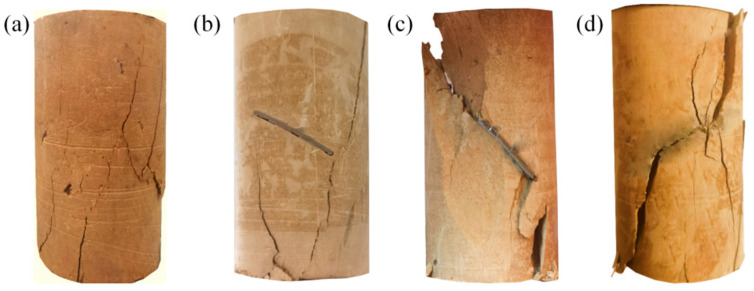
Damage patterns of silty mudstone specimens: (**a**) intact specimen; (**b**) FGDG–BFS material grouted specimen; (**c**) cement-based material grouted specimen 1; and (**d**) cement-based material grouted specimen 2.

Figure 11 shows the damage patterns of the intact specimens and the prefabricated crack silty mudstone specimens repaired with different materials. From Figure 11a,b, it can be seen that the damage patterns of the intact specimen and the specimen grouted with the FGDG–BFS material were the same. The damage pattern was longitudinal splitting damage along the center axis of the specimen, accompanied by crushing and dislodging of part of the block. It was demonstrated that the UCS of the grouted specimens using the FGDG–BFS material was close to that of the intact specimen. Because the shrinkage, UCS, and shear strength parameters of the FGDG–BFS material hardened slurries were similar to those of intact specimens, this avoided stress concentrations at the interface between the grouting material and silty mudstone due to the large differences in the engineering properties of the grouting material and the silty mudstone.

From Figure 11c,d, it is observed that the damage pattern of the cement-based material grouted specimens was shear slip damage along the direction of the prefabricated cracks. This was due to the significant differences in the engineering properties, such as the shrinkage, modulus of elasticity, and compressive strength, between the cement-based materials and silty mudstone. Under external loads, the interface between the cement-based material and the silty mudstone produced cracks first. This aggravated the cracking and damage of the silty mudstone. Therefore, compared with the traditional cement-based grouting material, the FGDG–BFS material had better improvement effects for the cracked silty mudstone. The above research results are similar to those of Jin et al. [45] and Le et al. [46], who found that a large difference between the physical properties of the grouting material and the target rock, such as UCS, would lead to cracks at the interface between the rock and the grouting material. The smaller the difference between the physical properties of the grouting material and the target rock, the more likely that secondary cracking of the grouted rock could be avoided. This proves the superiority of the FGDG–BFS material in repairing the cracked silty mudstone again.

## 4. Application of Grouting Material in Reinforcing Cracked Silty Mudstone Slopes

The purpose of this section is to examine the repairing effect of the developed grouting material on cracked silty mudstone slopes. Numerical simulations based on CDEM were employed due to the large volume and time-consuming nature of field tests and model tests. The advantage of CDEM over conventional finite element or discrete element software is its ability to well describe the elastic deformation and plastic deformation of soft rock [47]. The numerical analysis software (GDEM) adopted was developed by the Institute of Mechanics of the Chinese Academy of Sciences based on Continuous-Discontinuous Element Method (CDEM). More details about this software can be found in [48].

### 4.1. Validation of the Numerical Analysis Method

#### 4.1.1. Numerical Compressive Tests

**Figure 12:**
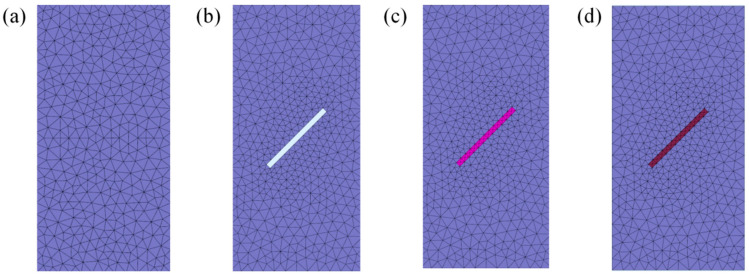
Numerical analysis model: (**a**) intact specimen; (**b**) 45° crack specimen; (**c**) cement-based material grouted specimen; and (**d**) FGDG–BFS material grouted specimen.

Unconfined compressive tests were simulated based on biaxial models (Figure 12). Intact specimens, specimens with different crack angles, specimens grouted with cement-based material, and specimens grouted with the FGDG–BFS material were considered. The thickness of the prefabricated crack was 3 mm and the width was 30 mm. A vertical load was exerted on the upper surface of the model at a loading rate of 0.002 mm/s. The material properties of silty mudstone, cement-based material, and FGDG–BFS material required in the numerical analysis are shown in Table 4. The strength parameters of the interface between the silty mudstone and cement-based material and the interface between the silty mudstone and FGDG–BFS material are presented in Table 5. The numerical results are compared with those obtained from laboratory tests to verify the numerical analysis method.

#### 4.1.2. Numerical Analysis Results

**Figure 13:**
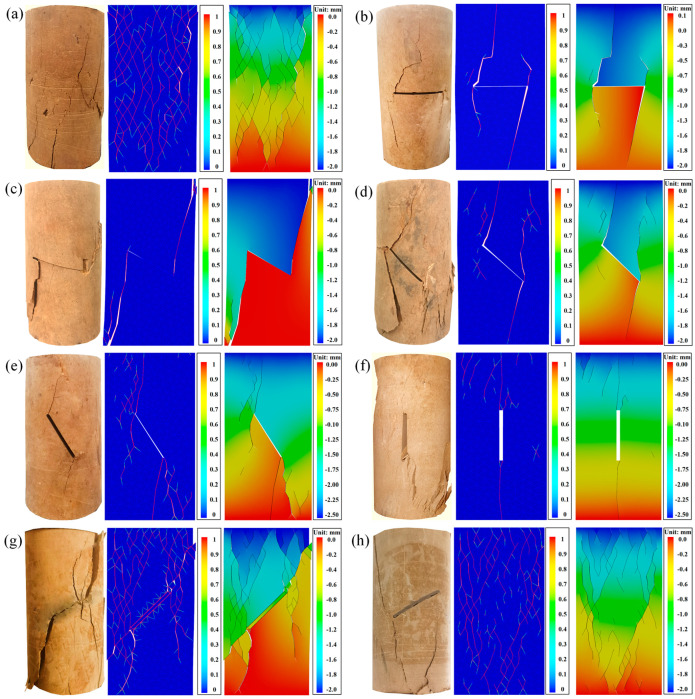
Unconfined compressive test crack evolution and vertical displacement: (**a**) intact specimen; (**b**) 0° crack specimen; (**c**) 30° crack specimen; (**d**) 45° crack specimen; (**e**) 60° crack specimen; (**f**) 90° crack specimen; (**g**) cement-based material grouted specimen; and (**h**) FGDG–BFS material grouted specimen.

Figure 13 shows the test damage patterns, numerically analyzed damage patterns, and vertical displacement cloud for intact specimens, specimens with different crack angles, and grouted specimens. In the legend of the numerical analysis damage mode figures, 1 represents the main crack and 0 indicates no cracking. As seen in Figure 13, the damage patterns of the specimens obtained from the tests and numerical analysis were almost the same. The difference between the test results and numerical analysis results using GDEM arose because the test specimens cannot be completely homogeneous and without impurities as in the numerical analysis model, but this does not affect the accuracy of the test results.

The specimens grouted with cement-based materials obtained from both tests and the analysis calculations exhibited shear slip damages along the direction of the crack of the prefabricated cracks, with vertical displacement occurring in the upper part of the specimens. The damage patterns and vertical displacements of the specimen grouted with the FGDG–BFS material and the intact specimen were similar. In summary, GDEM can well predict the mechanical behavior of silty mudstone with a high confidence.

### 4.2. Effectiveness of the Grouting Material in Reinforcing Cracked Silty Mudstone Slopes

The performances of slopes containing cracks, cracked slopes grouted with cement-based materials, and those grouted with the FGDG–BFS material were compared to investigate the improvement effect of the FGDG–BFS material.

#### 4.2.1. Numerical Slope Model

**Figure 14:**
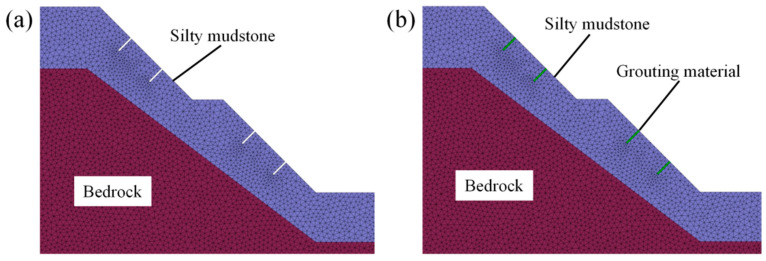
Numerical analysis modeling of slope: (**a**) crack slope and (**b**) grouting material grouted slope.

Two-dimensional numerical models of silty mudstone slopes were established by GDEM. The lower left point of the model was set as the origin with coordinates (0, 0). The height and length of the model were 40 m and 55 m, respectively. Four cracks were placed on the slope surface, as shown in Figure 14. The cracks were both at 90° to the slope surface, with a width of 0.2 m and a depth of 2.5 m. The mechanical properties (USC, tensile strength, etc.) of silty mudstone, cement-based material, and the FGDG–BFS material are referenced in Table 3. The interfacial parameters of the two grouting materials and silty mudstone are used as the values in Table 4. When a broken block sliding out of the slope area appeared, it was considered that the model reached a discrete stage and the calculation was finished.

#### 4.2.2. Results of Numerical Analysis

**Figure 15:**
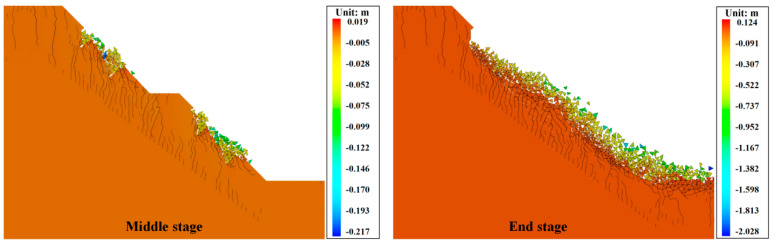
Vertical displacement cloud images of cracked slope.

Figure 15 shows the vertical displacements of the cracked slopes obtained using numerical calculations. In the middle stage, the silty mudstone in the vicinity of the initial cracks broke first. Afterward, the rock on the slope surface between the initial cracks broke up and slid down to the bottom of the slope. Subsequently, the silty mudstone on the slope surface between the initial cracks further broke up, and the silty mudstone on the cutting slope platform also broke. Eventually, the slope collapsed. Moreover, the depth of silty mudstone breaking was almost the same as the depth of the initial crack. Since the upper silty mudstone broke and collapsed first, the stress inside the slope was released. Therefore, although the silty mudstone below the depth of the prefabricated cracks also produced a large number of cracks, the slope did not further break and collapse. This indicates that cracks can lead to the collapse of silty mudstone slopes and increase slope destabilization.

**Figure 16:**
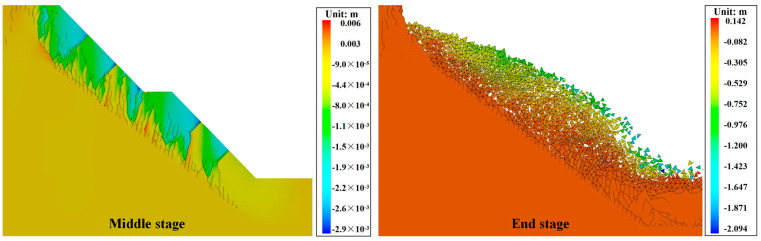
Vertical displacement images of silty mudstone grouted with cement-based material.

Figure 16 shows the vertical displacement cloud of the silty mudstone slope grouted with cement-based material. Compared with Figure 14, there was no damage to silty mudstone in the middle stage. This demonstrates that cement-based material can effectively reduce the risk of slope destabilization of cracked silty mudstone and improve the stability of the slope. However, cracks were generated at the interface between the grouting material and the silty mudstone, and the cracks extended downward. This phenomenon can be attributed to the large gap between the density and mechanical strength of cement-based grouting materials and silty mudstone, resulting in a low interfacial force between the two. Consequently, slope cracks continued to develop, which eventually led to the top-down breaking of the silty mudstone of the slope. Additionally, compared with the unrepaired slope, the internal stress of the slope was not released, which led to a higher degree of damage to the silty mudstone in the deeper part of the slope, causing the slope to undergo a deeper collapse.

**Figure 17:**
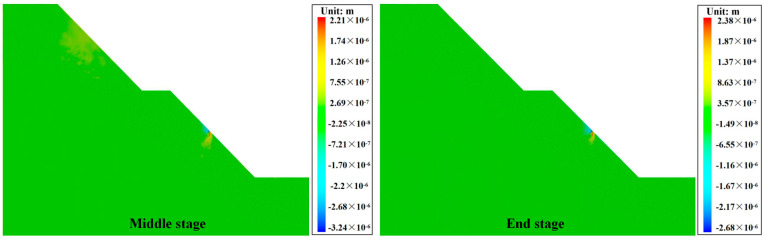
Vertical displacement images of silty mudstone grouted with FGDG–BFS material.

Figure 17 shows the vertical displacement cloud of the silty mudstone slope grouted with the FGDG–BFS material. Compared with Figure 16 and Figure 15, it can be observed that, at the same stage, there was no crack at the interface between the FGDG–BFS material and silty mudstone. This is because, compared with the cement-based material, the density, modulus of elasticity, and mechanical properties of the FGDG–BFS material were closer to those of the silty mudstone, and the interfacial mechanical properties of the two were better. Therefore, this FGDG–BFS material can better repair the weakening effect of cracks on silty mudstone slopes and improve the stability of the slope.

## 5. Conclusions

This study developed a new environmentally friendly industrial solid-waste-based grouting material (FGDG–BFS material). The optimal proportions of each component in the material were obtained through laboratory tests. In addition, the effectiveness of the FGDG–BFS material in enhancing the stability of cracked silty mudstone was investigated by GDEM. The following conclusions are finally drawn:

(1) The optimal BFS-to-FGDG ratio was 0.8:1, at which the UCS (15.6 MPa), tensile strength (0.47 MPa), cohesion (0.68 MPa), and internal friction angle (19.2°) of the hardened FGDG–BFS slurries cured for 14 d exceeded those of silty mudstone.
(2) The optimal dosage of SG was 0.4%. The softening factors, setting times, and diffusion diameters of the grouting material increased initially and then gradually leveled off with an increase in the dosage of SG. The lowest dosages at which these parameters reached a stable state were 0.3%, 0.4%, and 0.4%, respectively.
(3) The UCS, water resistance, and damage pattern of the FGDG–BFS material repaired specimens were similar to those of the intact silty mudstone specimens. In contrast, the specimens grouted with cement-based material exhibited a weaker performance and were damaged by shear slip along the interface between the grouting material and silty mudstone.
(4) Using GDEM to analyze the mechanical behavior and slope stability of silty mudstone is reliable. The numerical analysis results of the GDEM for four specimens closely matched the experimental results. The FGDG–BFS material effectively repaired the existing cracks and prevented re-cracking at the interface between the grouting material and silty mudstone, maintaining slope stability over a long period.

## Figures and Tables

**Table 1:** Basic physical parameters of raw materials.

Raw Materials	Particle Size Parameters/μm			Specific Surface Area /(m^2^·kg^−1^)	Apparent Density /(g·cm^−3^)
	D_10_	D_50_	D_90_		
FGDG powder	18.962	44.438	81.556	132	2.30
BFS powder	1.673	12.098	37.160	412	2.90

**Table 2:** Chemical composition of raw materials (%).

Raw Materials	CaO	SO_3_	Al_2_O_3_	SiO_2_	Fe_2_O_3_	K_2_O	MgO	Na_2_O
FGDG powder	31.12	44.44	0.77	0.57	0.22	0.17	0.22	0.07
BFS powder	43.19	0.10	12.60	25.52	0.49	-	15.21	-

**Table 3:** Component content percentage of FGDG–BFS grouting material.

No.	Water Content/%	BFS Content/%	FGDG Content/%
1	33.33	0.00	66.67
2	33.33	11.11	55.56
3	33.33	19.05	47.62
4	33.33	25.00	41.67
5	33.33	29.63	37.04
6	33.33	33.33	33.34

**Table 4:** Material properties of rock and grouting materials.

Material	Density /(g cm^−3^)	Tensile Strength /MPa	Cohesion /MPa	Internal Friction Angle/°	Elastic Modulus /GPa
Silty mudstone	2.4	0.66	0.62	18.7	15.1
Cement-based grouting material	4.9	3.75	3.5	27.5	35.0
FGDG–BFS grouting material	2.8	0.45	0.78	16.8	12.6

**Table 5:** Interfacial strength parameters.

Interface	Tensile Strength /MPa	Cohesion /MPa	Internal Friction Angle/°
Silty mudstone–cement-based material	0.39	0.35	12.4
Silty mudstone–FGDG–BFS material	0.57	0.69	18.4

## Data Availability

The original contributions presented in the study are included in the article material, further inquiries can be directed to the corresponding author.
